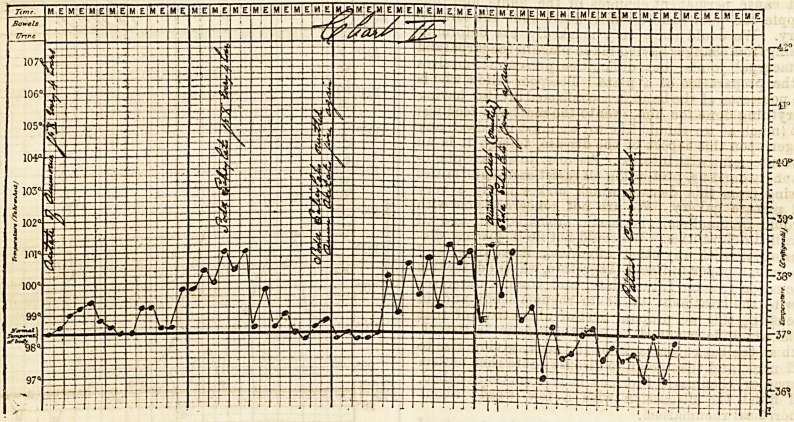# The Treatment of Rheumatism

**Published:** 1893-04-22

**Authors:** 


### The Hospital Clinic.

[*The Editor will be glad to receive offers of co-operation and contributions from members of the profession. All letters should be addressed to* The Editor, The Lodge, Porchester Square, London, W.]

### ROYAL SOUTHERN HOSPITAL, LIVERPOOL.

### Treatment of Rheumatism.

Of all diseases that this poor frail body is heir to, acute rheumatism is perhaps one of the most serious, not so much from the actual disease, as from the complications that may arise during the course of the disease. We grant that, with the present mode of treatment, they are not so common as formerly, but still they are quite frequent enough to warrant us in regarding acute rheumatism as a disease not to be trifled with. Who cannot call to mind some heart affection, either an endocarditis or a pericarditis, as a sequela or complication of rheumatism, and finally rendering the patient a burden to himself and his friends and unfit to carry out or follow the ordinary duties of life; and, again, how often do we see the acute variety of the disease lapse into the chronic variety, and cause permanent changes in the joints, and thus cripple the poor sufferer until death ends the terrible story. We could show very markedly the deformity arising from changes having taken place in the carpal and metacarpal joints, but difficulties present themselves in the preparation of a plate. The poor fellow who is the possessor of the hand is a confirmed invalid, having everything done for him, evento the matter of feeding; but (much as we should like to) we must not dwell any longer upon this aspect of the disease, though it is an aspect teeming with interest. As we write we have photographs around us, presenting all varieties of deformities arising from this disease, which makes really a very sad picture gallery. We are not concerned now with chronic rheumatism, rheumatic arthritis, &c., and all its allied deformities. We could not do it justice, discussing it with any other affection; it requires, and deserves, a paper to itself. Therefore, we will defer this discussion to some future date, and confine ourselves now to the treatment of acute rheumatism; and in discussing this we propose first of all to treat of that form which is confined to certain organs or tissues, such as the back (lumbago) or the neck, giving rise to stiff neck, and then go on to discuss acute rheumatism proper or rheumatic fever, as it is sometimes called, and finish the discussion with a few words about gonorrhœal rheumatism.

Well then, with reference to the treatment of lumbago, stiff neck, &c. Rheumatism in this form presents, as a rule, no constitutional symptoms except the pain at the seat of localisation. We may get the sciatic nerve affected, giving rise to sciatica, or, as we mentioned, the muscles of the back, giving rise to lumbago, or the sterno-mastoid, causing stiff neck, and in all these affections we combine local and general treatment; the application of blisters to the affected part, or the actual cautery drawn once or twice over the part (in this case we, of course, give a little anæsthetic), and in the case of the sciatic nerve, forcible flexion of the thigh upon the pelvis, with the extension of the leg upon the thigh, causing a stretching of the nerve, or puncturing the nerve in several places along its course with a long, sharp needle; or, again, injecting morphia hypodermically (one-sixth of a grain) into the nerve. One or other of these methods we generally find relieve, but they are usually combined with a little internal medicinal treatment; a favourite prescription is the following: Soda salicyl, gr. vii.; pot iodid, gr. iv.; Tr gent. co. mx.; aq. chlorof. ad ℥i., and this mixture to be given three times a day; the salicylate and iodide we hope has a specific action on the disease, and the gentian will act as a tonic. The patient is allowed to be up and about, except when any local application has to be applied, or any raw surface, such as that arising from a blister, or the actual cautery to be dressed. The diet is not so carefully looked after in this form of the disease as in the acute variety. We find a wholesome, nutritious, and liberal diet necessary, for unless this is carefully attended to the patient soon begins to lose flesh and run down the hill; so we allow them fish, game, and meat of any sort, the meat, of course, only once a day, except, perhaps, bacon for breakfast as well as the midday meat meal. Eggs are allowed for tea or supper, and milk—a pint, or pint and a half—during the day. A little stimulant is even allowed, especially where the patient is very much "below par." Bitter beer or stout are the ones generally chosen or prescribed, and to complete the treatment, if the weather is anything like favourable they are sent away into the country, where, after some few weeks' residence a cure, for the time being, results. We say for the time being, because these cases of rheumatism are so apt to recur on the slightest provocation, even a slight chill, or indiscretion of diet, or anything that tends to lower the vitality of the individual, bringing back the old malady.

We now come to the treatment of perhaps the most important rheumatic affection, we mean acute rheumatism, or rheumatic fever, as it is sometimes called. It is important because of the serious complications that may arise during the course of the disease. Happily we do not see them so frequently as used to be the case in former days, when acute rheumatism was looked upon, one might almost say, as an acute specific disease, to run its course in six weeks, and these six weeks were necessary for the cure. No medicines were ever given with the hope of cutting short the disease; perhaps a little mint water was prescribed to satisfy the cravings of the patient for medicine. Now we can fortunately (owing to the benefits of sodæ salicylatis) tell a very different tale; we have in salicylate of soda almost a specific for the disease. What a marked effect it has on the temperature the following charts will show. The second chart is especially interesting, as it shows particularly the influence of soda salicyl over the temperature, it being left off two or three times and acetate of ammonium given in its stead, the temperature immediately commences to rise on the salicylate being left off. And again, how valuable this drug is in diminishing the pains of acute rheumatism, pains that are so severe that they would otherwise require a strong opiate to relieve. Another benefit, we think, to be derived indirectly from soda salicylate is that, owing to its cutting short the disease, it prevents, in a very great measure, cardiac lesions. The line of treatment, then, that is carried out at the above hospital is that, directly on admission, the patient is put to bed between blankets. Blankets are used to prevent any chance of chills that might possibly arise owing to the saturation of the sheets with the moisture from the patient's body, which is sometimes very profuse in these cases, and a flannel night garment is also put on. These continue the salicylate in diminished (say once or twice a day as before mentioned) doses, even though the temperature has been normal, because if left off too soon, a relapse is sure to occur, and when we think it safe to leave it off altogether, the temperature is watched very carefully for some days afterwards. We find that if the bowels are acting regularly and freely we do not get so readily the symptoms of salicylic poisoning; we mean deafness and singing in the ears; therefore the bowels are kept freely open, either with col. et hyoscy. pills every other night if necessary, or a dose of mist. sennæ co.; by these means we obviate to a great extent the evil effects of salicylate. Tonics, such as iron and quinine, are prescribed as the patient convalesces. The diet is a milk diet, consisting of milk puddings, any variety, milk, and tea and bread and butter; when the patient is convalescent the ordinary articles of food are allowed. The joints, if very painful, are painted with glycerine and belladonna and wrapped round with cotton wool, and fastened on with flannel bandages; the joints are protected from pressure of the bedclothes by using cages to suspend the clothes. Should any discomfort arise from the profuse perspiration, we find that sponging with tepid vinegar and water greatly precautions thus seal them against any further chilling of the body. Perhaps the patient has got his attack of acute rheumatism through sleeping in damp sheets, and surely, if we allow him again to lie in sheets that will soon become saturated with the moisture that he has already absorbed, in the shape of acute rheumatism, from the previously damp sheets, we should be adding "fuel to the fire." Having accomplished so much, we immediately commence the soda salicylate, and the idea is to saturate the system with the drug, and with this view it is given every two hours for four doses, and then every four hours, and it is persevered with at this dosage through the course of the disease, except when any bad symptoms, such as singing in the ears or deafness, come on; the dose is then diminished in frequency. As the disease subsides the salicylate is gradually diminished, first to three times a day, then twice a day, and finally once a day, after which it is left off altogether. The following is the prescription as generally used: Sodæ salicylatis gr. x., spr. chlorof m. x., aq. ad. ℥i. The salicylate will probably have thus been taken for about a fortnight, though the temperature has remained normal for possibly a week, but we find it very necessary to continue this. relieves this. Should any cardiac complication arise such as pericarditis, the præcordia is blistered freely with the hope of stopping the pericardial inflammation; should unfortunately this pericarditis go on to effusion, the question comes of aspirating the pericardial cavity, and if dyspnœa or orthopnœa are present, with no sign of absorption of the fluid, we feel called upon to aspirate, and this is done with all the antiseptic precautions that are observed in aspirating a thorax which we mentioned in a previous paper. The needle is taken and gently pushed in, or, which is perhaps better, an incision is made through the skin and subcutaneous tissues until one almost reaches, or quite reaches, the parietal pericardium) when the needle can be quite easily pushed in without much fear of wounding the heart. The point we generally choose for the incision is between the fourth and fifth ribs, close to the sternum, being careful to avoid the internal mammary artery. The toilet of the pericardium is completed with that care that we insisted upon in thoracic aspiration or abdominal aspirations. Should, unfortunately, we meet with pus in the pericardium, we (if the abscess is not pointing), open in the same spot as for aspirating, and insert a small drainage tube. It is particularly in these cases that stimulants are called for, and we prescribe them accordingly. The prognosis of pus in the pericardium is about as bad as it can possibly be, and we feel that in opening the pericardium for pus we have come to the *dernier ressort* and that only by a miracle can the patient survive. With reference to endocarditis we find that there is no decided indication for any special line of treatment; absolute rest in bed is, of course, very essential, and opium in these cases, as well as in pericarditis, is of inestimable value, and we prescribe the pill opii gr. a-quarter or half, or pulv. opii, combined with one-sixth or one-eight grain of calomel, and this to be taken every four hours; it quiets the patient and relieves the pain.

Another complication which we must mention before closing is hyperpyrexia (a temperature above 105 deg. or 106 deg.), and this requires immediate attention. We generally sponge the body with first tepid water, reduced finally to ice cold, continued until the temperature has dropped some three or four degrees. The cold bath we have practically discontinued, we found the shock so great during and after the immersion.

In conclusion, a few words about gonorrhœal rheumatism. We find in these cases the ankle and knee joints are peculiarly susceptible to the influence of the poison, and that the disease has a great tendency to leave behind stiff joints, also a great tendency to the occurrence of suppuration in the joint or joints affected. We cannot, unfortunately, say very much in favour of drugs, or treatment generally, for the relief of the stiffness that results from this form of the disease. Our experience is that the majority of cases are crippled more or less for the remainder of life. We find that improving the general condition by the use of tonics, and massage systematically practised on the affected joints, gives as great relief as any method of treatment, and the removal to a warm southern clime will help very materially.

## Figures and Tables

**Figure f1:**
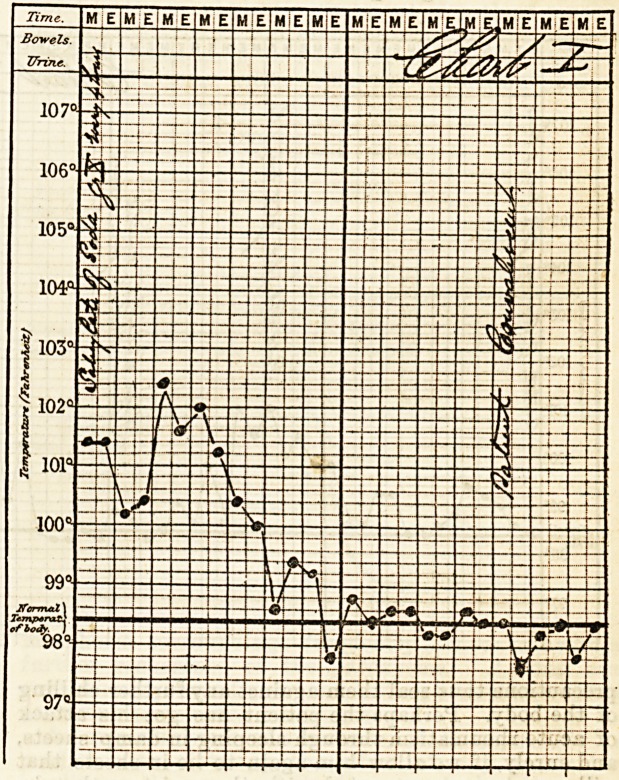


**Figure f2:**